# Application of national pollutant inventories for monitoring trends on dioxin emissions from stationary industrial sources in Australia, Canada and European Union

**DOI:** 10.1371/journal.pone.0224328

**Published:** 2019-10-25

**Authors:** Khushbu Salian, Vladimir Strezov, Tim J. Evans, Mark Taylor, Peter F. Nelson

**Affiliations:** Department of Environmental Sciences, Faculty of Science and Engineering, Macquarie University, Sydney, NSW, Australia; Institute for Advanced Sustainability Studies, GERMANY

## Abstract

Industrial sources, including iron ore sintering, municipal waste incineration and non-ferrous metal processing have been prominent emitters of dioxins to the environment. With the expanding industrial sectors, many international conventions were established in order to reduce the emission of dioxins in the past two decades. The Stockholm convention, a global monitoring treaty, entered into force in 2004 with the aim to promote development of strategies to reduce or eliminate dioxin emissions. According to the convention, parties are required to develop national inventory databases to report emission levels and develop a national implementation plan (NIP) to reduce further dioxin emissions. In order to understand the trend of dioxin emissions since 1990s this study provides a comparative assessment of dioxin emissions from different industrial sources by deriving emission data from the national inventory databases of Australia, Canada and the 28 European countries (EU-28). According to the data collected, iron and steel production and electricity generation were the highest emitters of dioxins in 2017 for Europe, Canada and Australia, when compared to other stationary industrial sources. The change in the trend of dioxin emissions from the iron and steel industry and the public electricity sector was also assessed. The emission of dioxins during 1990–2017 from both iron and steel production and electricity generation revealed a relative decreasing trend, except for Spain and Italy who showed higher level of emissions from iron and steel production in 2017. Furthermore, comparing emission data for metal production revealed that the blast furnace process was the prominent emitter of dioxins comparing to electric arc furnace process. Further investigation was performed to compare the amount of dioxin emitted from three different fuel types, black coal, brown coal and natural gas, used for electricity generation in Australia. The study showed that dioxin emissions from brown coal were higher than black coal for the last two years, while power production from natural gas emits the lowest amounts of dioxins to the environment.

## Introduction

Dioxins are unintentionally produced persistent organic pollutants (POPs), emitted in relatively low concentrations that persist in the environment for many years and have the tendency to bioaccumulate in the fatty tissues of living organisms and the environment [1]. Polychlorinated dibenzo-*p*- dioxins (PCDDs) and polychlorinated dibenzofurans (PCDFs) are a group of aromatic hydrocarbons, produced largely by various anthropogenic combustion processes in the presence of a chlorine source [2, 3]. The position and number of chlorine atoms surrounding the two benzene rings depict the physical, chemical and toxicological properties of these persistent organic compounds [2]. 2,3,7,8- Tetrachlorodibenzo-*p*-dioxin (TCDD), is the most toxic congener known to cause extreme toxicological impacts, such as carcinogenicity, teratogenicity, modulation of the immune system and tumour promotion [2, 4, 5]. TCDD is classified as group 1 carcinogen by the International Agency for Research on Cancer (IARC)[6]. Emission of dioxins from industrial sources is a major issue, as these compounds are generated as unwanted by-products from different processes, including municipal waste incineration, electricity generation, petroleum refining, iron ore sintering and other non-ferrous metal processing [7–9].

In order to achieve a better understanding of dioxin emissions to air from industrial sources, many countries have compiled national air pollution inventories that report the amount of released pollutants per annum, including release of PCDD/Fs emissions [10,11]. Toxicity equivalents (TEQ) are used to report emissions of dioxins in the national inventory reports, using the international toxicity equivalency (I-TEQ) established by the NATO/CCMS Working Group [12, 13]. The national pollutant inventories can serve as a database to assess the state of dioxin emissions on a national level and determine the effect of different international conventions on emission reduction, which have not been conducted before.

The United Nations Economic Commission for Europe (UNECE) implemented the Convention on Long-Range Transboundary Air Pollution (CLRTAP), which entered into force in 1983 [14]. The aim of the convention was to lay down general principles and set up an institutional framework for abatement of air pollution. Thirty-two countries signed the UNECE convention in the pan-European region, in the United States and in Canada. In 1998, protocols were established to reduce persistent organic pollutants, with an objective to control, reduce or eliminate discharges, emissions and losses of persistent organic pollutants (POPs)’ [15]. In Europe, municipal solid waste incineration was the highest emitter of dioxins in the 1980s and had remained the focus of research and public as well as political concern for more than ten years [7]. According to the final results obtained from the European Dioxin Air Emission Inventory in 2004, iron ore sintering was considered as the most important source of dioxin emissions, followed by municipal waste incineration [10]. Other sources that showed significant dioxin emissions were hospital waste incineration, secondary zinc recovery and electric arc furnaces (EAFs). The two processes (sintering and EAF) involved in iron and steel production contributed to the emission of dioxins in Europe. Extensive research has been performed in order to understand the formation and development of techniques to reduce dioxin emissions from iron ore sintering and steel making [2, 7, 16–19]. Qian et al., (2018) provided an overview of the production mechanism of dioxins and also discussed various measures to reduce dioxin formation during the iron ore sintering process. Three approaches for source control were discussed, including process control and end treatment, that can be adopted to reduce dioxin emissions from iron ore sintering plants [4].

Canada ratified CLRTAP in 1998, with the first Dioxins, Furans and Hexachlorobenzene Inventory of Release being published in January 1999 [20]. The inventory report prioritized six sectors that accounted for about 80% of national emissions: waste incineration (municipal solid waste, sewage sludge, hazardous and medical waste), pulp and paper boilers, iron sintering, residential wood combustion, electric arc furnace and conical municipal waste combustion. This report led to the development of the Canada Wide Standards (CWS) for dioxins and furans, with an ultimate goal of elimination and pollution prevention by reducing the releases to the environment or avoiding further production of dioxins [20].

In May 2004, a global monitoring treaty termed Stockholm Convention on Persistent Organic Pollutants entered into force. Initially, only 12 POPs were on the list and recommendations were developed on the basis of available information to implement international actions [21]. Dioxins were listed under the by-products category (Annex C, Part I) known as the unintentionally produced POPs. According to the convention, parties are required to track the emission levels and prioritize sources of unintentionally produced POPs, in order to develop strategies that aid in reducing the total releases derived from anthropogenic sources [12, 22]. The Stockholm Convention developed Best Available Techniques (BAT) and Best Environmental Practices (BEP) for the reduction and/or elimination of dioxin emissions. The guidelines on BAT and BEP are provided for new and existing sources of unintentionally produced POPs. Some of the BAT for reduction of dioxins from exhaust gas cleaning processes consist of sorption processes, dust separation, rapid quench systems, scrubbing processes and catalytic oxidation [23]. For waste incineration, some of the BEP include on-site procedures, such as handling residues, incinerator management and operation practises, waste inspection and handling, along with appropriate off-site procedures such as waste management [23]. Each party, under the Convention, is required to develop an inventory report that provides continuous data on the reduction of emissions. This was achieved by the establishment of the Global Monitoring Plan (GMP) [12, 24]. Furthermore, parties are required to provide a National Implementation Plan (NIP) outlining the actions adopted and propose future strategies for the reduction of emissions. Canada and most Member States of the European Union have ratified and entered the Stockholm Convention, except for Italy [25, 26].

The Stockholm Convention was ratified by Australia in May 2004 and came in effect in August 2004 [27]. According to the Sources of Dioxins and Furans in Australia (2002), report developed by the Australian Environment Protection Group (EPG), 75% of the total dioxin releases were from wildfires and biomass combustion from prescribed burning [28]. Secondary sources were sinter production, coal combustion, residential wood combustion and industrial wood combustion. In October 2005, Australian Environment Protection and Heritage Council (EPHC) released the National Action Plan for Addressing Dioxins in Australia in order to reduce dioxin emissions according to Article 5 of the Stockholm Convention [11]. According to this report, uncontrolled combustion (accidental fires, waste burning, prescribed burning and wildfires) contributed to 70% of dioxin emissions and the second highest emitters were metal smelting (iron and steel, zinc and aluminium) and fossil fuel power generation. The National Implementation Plan developed in 2006 by the Australian Department of Environment and Heritage, provides detailed information about various BAT and BEP discussed in the Stockholm Convention, which were adopted by the Australian Government to reduce or eliminate persistent organic pollutants [27]. Actions undertaken by Australia to meet the obligations under Article 5 of Stockholm Conventions, are outlined in the 2005 National Action Plan for Addressing Dioxins (NAP) [11]. The NAP provides details about various actions undertaken to minimise or eliminate dioxin releases to air, water, soil, sediment, biota and wastes. Some of the actions included adoption of a guideline emission level (0.1 ng TEQ/m) for all existing and new combustion facilities, remediation of sites contaminated with significant levels of dioxins, and undertake extensive research to improve knowledge on sources and exposure risks of dioxins in soils [11].

Inventories from many countries in the early 1990s were often out of date, incomplete and had no uniform structure. In addition, many countries lacked financial and technical aspects required to measure dioxins from all potential sources [12]. In 2003, United Nation Environment Programme (UNEP) developed the ‘Standardized Toolkit for the Identification of Quantification of Dioxin and Furan Releases’ (referred to as ‘Toolkit’). Under the obligations of the Stockholm Convention, many parties (countries) have been using the Toolkit methodology without dioxin analysis or collection of samples [12, 29]. Release estimates were calculated by multiplying process-specific default emission factors, provided by the Toolkit, with national activity data. Since 2005, the Toolkit has been revised to provide a bridge for developing countries in order to verify their emission factors and also emphasise on major sources which had limited data available [30].

The aim of this work is to investigate the release of dioxins in the past years (1990–2017) by comparing emission data from different stationary industrial sources. Emissions from European Union, Australia and Canada were compared in this study, as these selected countries have accessible national pollutant inventories. Iron and steel production has been the primary producer of dioxins in the past two decades and many strategies and technologies have been implemented in order to reduce the emission levels. Electricity production is one of the largest stationary industrial sectors that requires further assessment. Thus, the study discusses the change in the emission level of dioxins since 1990s from iron and steel production and electricity.

## Materials and methods

In this work, the emissions of dioxins from industrial operations were assessed for 30 countries, which include Australia, Canada, and 28 countries of the European Union (EU). The data for assessment of dioxin emissions from various industrial sources were collected from the national inventory databases. For Canada and the European countries (EU-28), data were extracted from the WebDab database, which is an emission database of European Monitoring and Evaluation Programme (EMEP) [31]. EMEP is responsible for evaluation and monitoring of the long range transmission of air pollutants in Europe. For Australia, the National Pollutant Inventory (NPI) database was used [32]. The Toolkit methodology is used by both national inventories to compile data on dioxin emissions from various sources [33, 34]. Some limitations include lack of linkage between local and regional environmental monitoring with the inventory data, better documentation of emission factors, lack of comparative analysis between inventory data and life cycle impact assessment and changes in site operations are not quantitatively analysed [35, 36, 37]. For this study, the two inventories were selected as they are publicly accessible and they provide an overview of the emissions, which can be compared.

Stationary industrial sources, which manufacture products with economic market value were selected for this study. Emission data from 1990–2017 were extracted from the WebDab database for Europe and Canada and from 1999–2017 from the NPI database for Australia.

For the purpose of analysing dioxin emissions per tonne (t) of metal produced, data of the total metal produced annually by each country was extracted from the Steel Statistical Yearbooks (1990–2017) published by the World Steel Associations [38]. In order to assess the amount of dioxin emitted from electric arc furnace and blast furnace production routes, Australian based iron and steel making companies were selected. Whyalla Steelworks is located approximately 400 km north–west of Adelaide and produces iron using the blast furnace route. Three other onesteel facilities situated in three locations (Rooty Hill-Sydney, Laverton-Melbourne, and Waratah-Newcastle), produce raw steel using electric arc furnace (EAF) [39]. Data on metal production per annum of these industries were extracted from the annual reports (2005–2015) of the parent company Arrium Limited, as the reports are only available untill 2015 [40]. Dioxin emissions from these two companies were collected from the National Pollutant Inventory (NPI) database for the respective facilities.

For each country, data of the total electricity generated annually was extracted to assess dioxin emissions per terajoules (TJ) of electricity produced. For all the European countries, the Eurostat database was used to collect data on total electricity produced annually (data available from 1990–2016) [41]. Data for Australia was extracted from the Australian Energy Update 2018 report, developed by the Australian Government (Department of Environment and Energy) [42]. To analyse the amount of dioxin emitted from various Australian power stations that use black coal, brown coal or natural gas, emission and electricity generation data were collected for six black coal fired power stations (Energy Australia- Mt. Piper, Macquarie Generation-Bayswater, NRG Victoria- Gladstone, Stanwell Cooperation- Stanwell, Electricity Generation and retail Cooperation- Muja and Kwinana), six natural gas fired power stations (Energy Australia Holdings Limited- Tallawarra, Stanwell Cooperation- Mica Creek, Synergen Power- Dry Creek, ALG Energy–Torrens Island, Ecogen Holdings- Newport, Origin Energy- Mortlake) and three brown coal fired power stations (AGL limited- Loy Yang A, IPM Australia- Loy Yang B, Energy Australia Holdings- Yallourn). The selected power stations represent typical coal fired power station technologies which are equipped with particle capture devices, such as fabric filters or electrostatic precipitators, but exclude off-gas scrubbing and activated carbon injections. Electricity generated by each of these power stations was extracted from Electricity Sector Emissions and Generations report produced by the Australian Government (Clean Energy Regulator) [43]. Dioxin emission data for each power station was collected from the NPI database.

## Results

**Fig 1:**
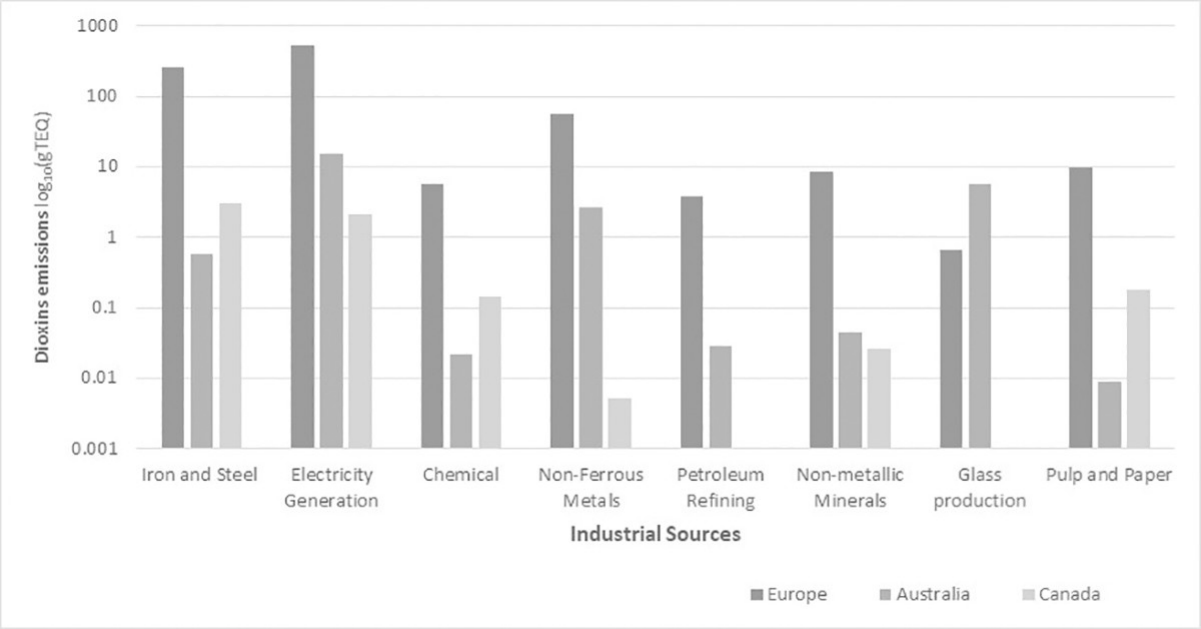
Dioxin emissions from major stationary industrial production sources for 2017.

Fig 1 shows the amount of dioxins emitted from a range of stationary industrial sources for 2017. The actual emission values were substantially different, therefore, the values were converted to log scale. The emission rates from electricity generation are the highest for Europe, followed by iron and steel industries and non-ferrous metal production. For Australia, electricity generation is the highest emitter of dioxins. The glass production industry in Australia is the second prominent emitter of dioxins. Emission from the iron and steel industry is the highest in Canada, with electricity generation being the second highest source.

### Emission from iron and steel

**Fig 2:**
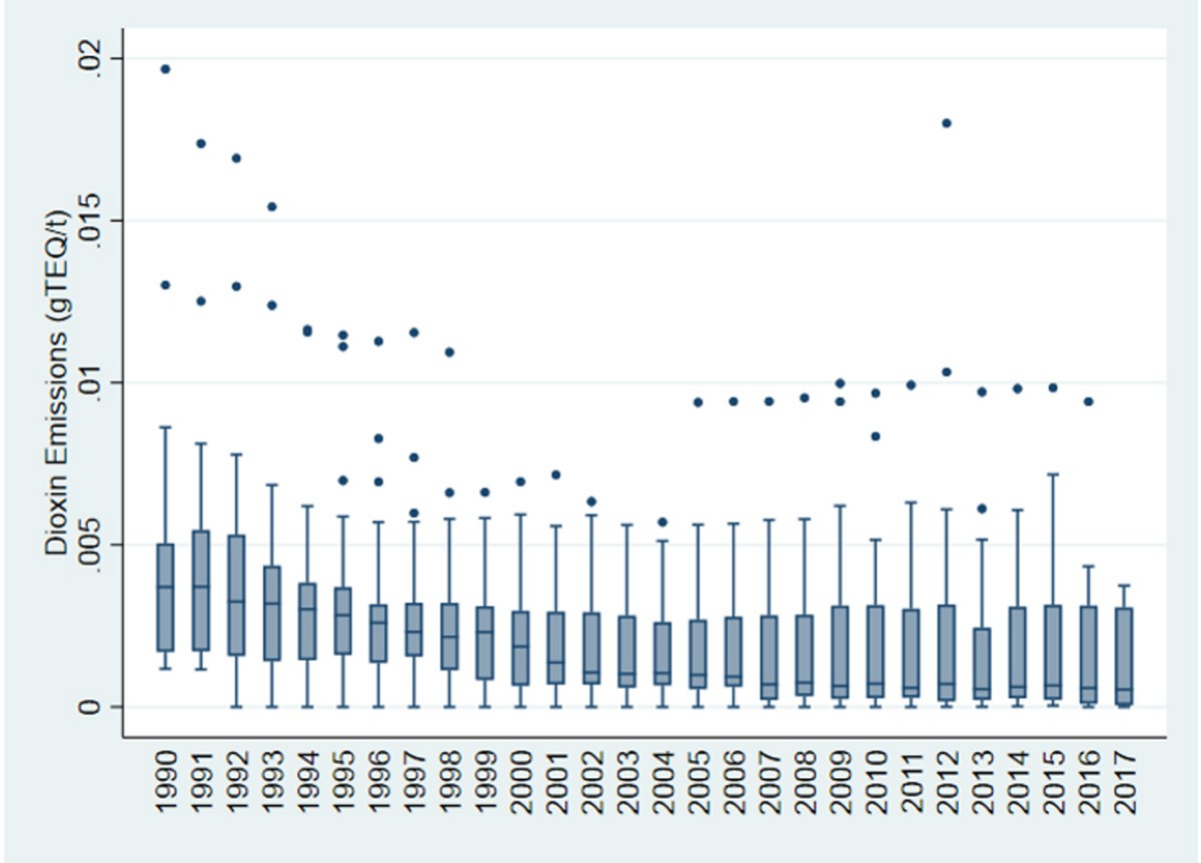
Dioxin emission per tonne of ferrous produced from 1990–2017.

**Fig 3:**
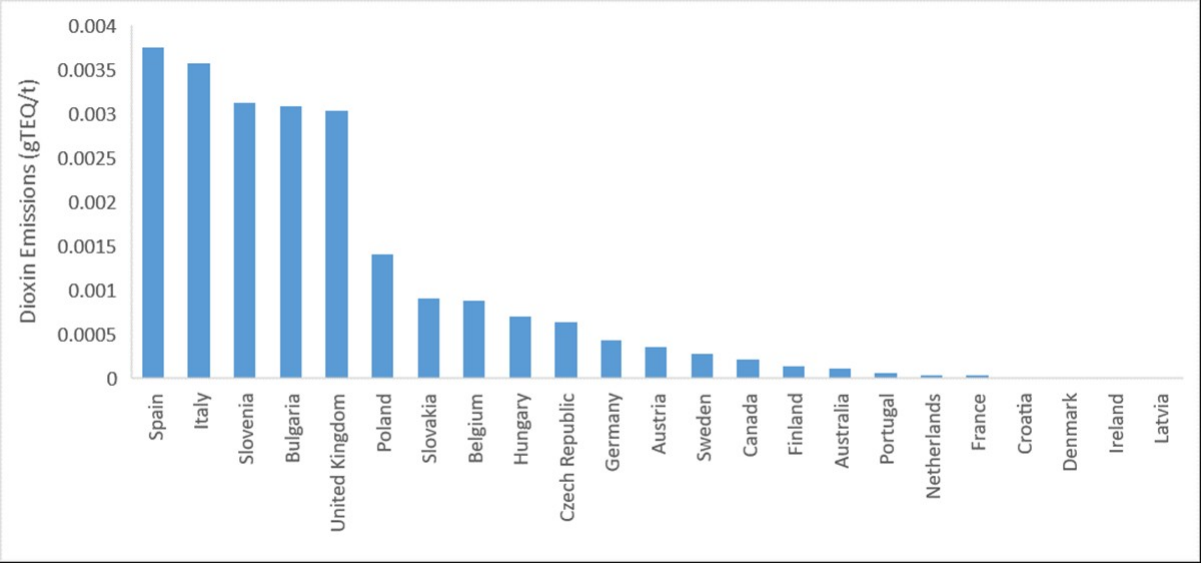
Dioxin emission per tonne of ferrous produced in Australia, Canada and all European Countries (EU-28) in 2017.

Assessing the amount of dioxin released per tonne of metal produced, revealed a decreasing trend of dioxin emissions over the period of 1990–2017 (Fig 2). The graph accounts for data derived for Australia, Canada and EU-28 countries only. The ends of the box represent the upper and lower quartiles (second highest and lowest values for that year), which is used as a measure of data spread for each year excluding the outliers (dots on the graphs). The figure also identifies the outliers in the data sets. The median (mean of two middle numbers) values (horizontal line inside the box) show a gradually decreasing trend in the emission rates of dioxins per tonne of metal produced. Emission rates in 2017, as shown in Fig 3, were the highest for Spain, Italy, Bulgaria, Slovenia and United Kingdom.

According to the Informative Inventory Report of Spain (2017), there was a decline in the emission of dioxins from iron and steel production between 1990–1998 [44]. Due to an increase in the production of steel in electric furnaces from 1999–2007, there was progressive rise in the emission rates of dioxins in Spain. Also, there was a sharp decline in dioxin emissions in 2009 and 2012, as the production of steel in electric furnaces decreased [44]. The report directly relates the production of steel in electric arc furnaces to the variations in emission of dioxins over the years. According to the report, the rise in emission of dioxins from iron and steel industry in 2017 is due to the increase in the production of pig iron by 8.39%, sinter production by 6.35% and steel production by 7.73% [44].

For Italy, the national dioxin emissions decreased (equal to 43%) from 1990 to 2017, with a noticeable decline occurring between 1995 and 2004 [45]. This was largely due to the inculcation of abatement strategies in one of the Italian steel production plants, which contributes to more than 80% of steel production of the country. Installation of double filtering system ESP (electrostatic precipitator), injections of urea that stabilise the metals responsible for dioxin formations, along with reduction of amount of chlorine in the charge are some of the abatement systems employed by the Italian steel plant [45]. However, emissions specifically from iron and steel industries have increased (30%), since 1990 in Italy. Emissions from production processes account for 28% increase from 1990–2017, which is due to the installation of electric arc furnaces for iron and steel production [45].

**Fig 4:**
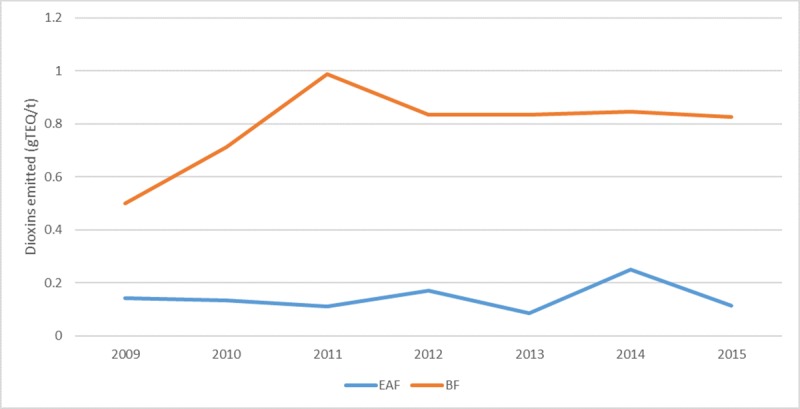
Dioxins emitted by blast furnace (BF) and electric arc furnace (EAF) ferrous production companies in Australia from 2009–2015.

Dioxin emissions from crude metal producing companies located in Australia were further compared to assess the differences in emission rates between the blast furnace (BF) production route and electric arc furnace (EAF) based steelmaking. Since EAF is a recycling method, which uses ferrous scrap to produce crude steel, the amount of dioxin emitted from the three EAF facilities considered in this work was significantly lower when compared to the emissions reported from the BF production route, which uses iron ores and coals to produce crude steel (Fig 4). It should be noted that Whyalla steelworks is an integrated steel mill, which produces both iron and steel products. In 2014, EAF contributed to 26% of the world’s steel production [46], thus, with the expansion of the EAF process in the future, the rate of dioxin emissions is expected to also decrease. The National Action Plan for addressing dioxins in Australia provides a guideline emission level of 0.1 ng TEQ/m^3^ for all existing combustion facilities and also for new facilities [11]. The emission of dioxins from all the industrial facilities in Australia for 2017 totals 32.62 g TEQ. Therefore, new strategies and improved guidelines are required to reduce the emission of dioxins with increasing industrial production.

### Emission from public electricity

**Fig 5:**
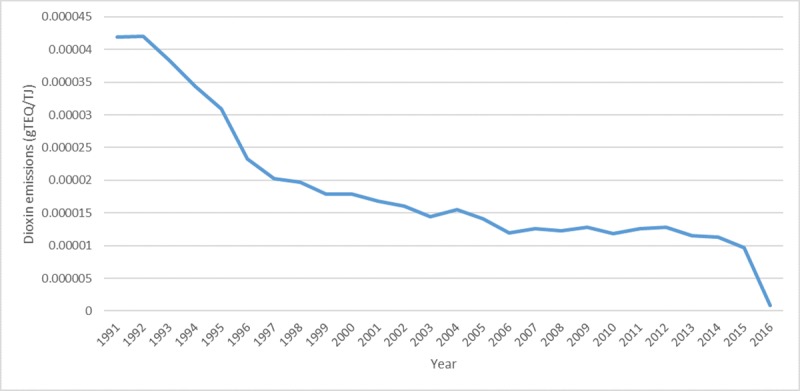
Dioxins emitted per TJ of electricity generated from 1990–2016.

The level of dioxins emitted per terajoule (gTEQ/TJ) for Australia and the EU-28 countries is shown in Fig 5. The graph depicts a decreasing trend of dioxin emissions per TJ of electricity produced from 1990–2016. According to the overall electricity production data for EU-28 countries 7.8×10^6^ TJ and 9.9× 10^6^ TJ of electricity was generated in 1990 and 2015 respectively, which shows that the production has increased by 27.3% [47]. Thus, with increasing electricity production, dioxin emissions have been reduced over the years.

**Fig 6:**
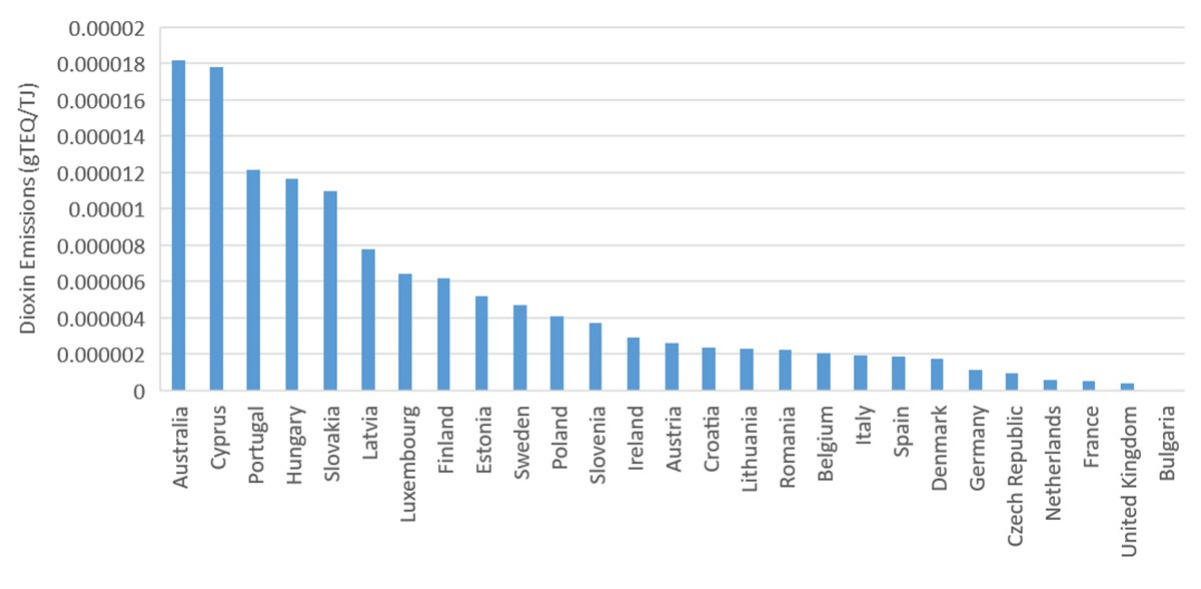
Dioxins emitted per TJ of electricity generated in all EU-28 countries and Australia in 2016.

Assessment of dioxins emitted from each country revealed that Australia is the highest emitter of dioxins per TJ of electricity produced in 2017, followed by Cyprus and Portugal (Fig 6). According to the NPI data, Hazelwood power station based in Victoria, Australia showed the highest emission of dioxins (13%) in 2015. However, this brown coal fuelled power station was decommissioned in 2017 [48]. Bayswater power station was the highest emitter of dioxins (12%) in 2016, which is a black coal-fired power plant based in New South Wales, Australia, followed by Yallourn (9%) located in Victoria, Australia, which is a brown coal fuelled power station.

**Fig 7:**
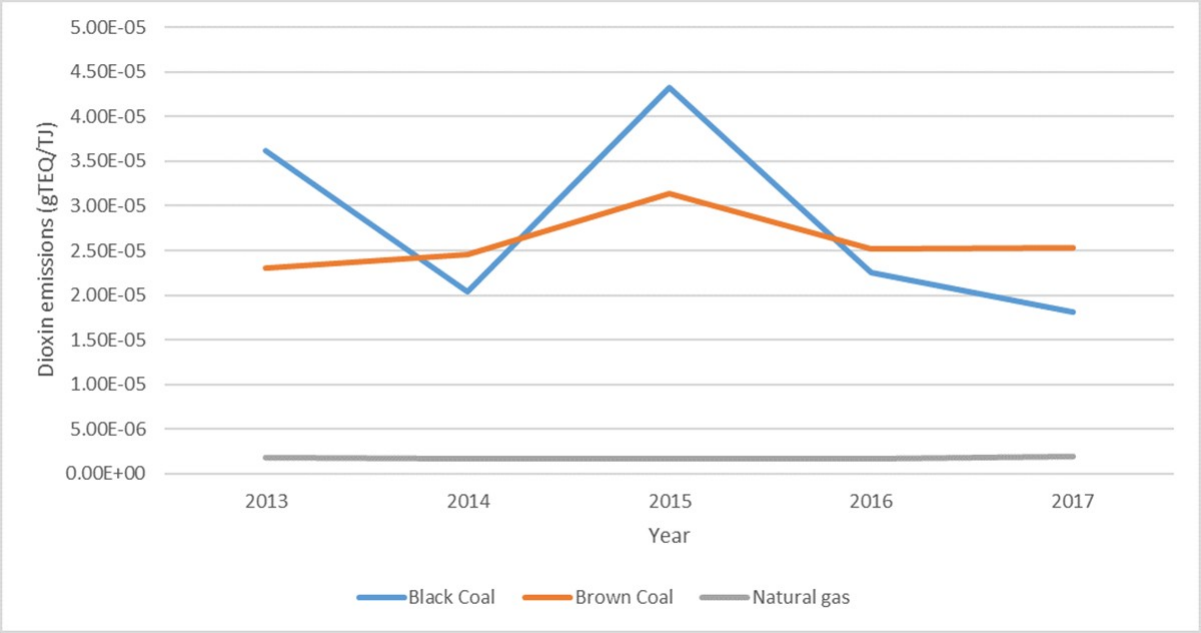
Dioxin emissions from power plants using black coal, brown coal and natural gas.

To determine the impact of the fuel source on dioxin emissions, 2013–2017 data from six black coal, six natural gas and three brown coal fired power plants in Australia were compared, as shown in Fig 7. The results show that the level of dioxins emitted from gas fired power plants are significantly lower compared to brown and black coal power stations. The emission levels between electricity production from black and brown coal intermittently change over the years, with brown coal exhibiting higher dioxin emission levels per amount of electricity produced in the last two years. Bayswater, a coal fired plant located in Muswellbrook in the upper Hunter Valley of NSW, is the highest emitter of dioxins as compared to the other black coal fired power plants from 2013–2017. The emission from Yallourn power station, that uses brown coal located in Latrobe Valley in Victoria, was higher than Bayswater in 2017.

## Conclusion

This study provides a comparative assessment of dioxin emissions from different stationary industrial sources for major countries which include Australia, 28 European (EU-28) countries and Canada. According to the 2017 emission data acquired from the respective national pollutant databases (NPI and WebDab), electricity generation was the highest emitter of dioxins in Europe and Australia. Iron and steel industry was the prominent emitter of dioxins in Canada, followed by electricity generation. According to the Chinese National Implementation Plan for the Stockholm Convention (2007), iron ore sintering is the highest (30%) emitter of dioxins as compared to other chlorinated POPs [49].

Furthermore, the study assessed the change in the level of dioxins emitted from iron and steel industries (1990–2015) and public electricity generation (1990–2016). The assessment showed that dioxin emissions per tonne of produced metal reduced substantially for Australia, Canada and EU-28 countries. Emissions from Spain, Italy, Bulgaria, Slovenia and United Kingdom from iron and steel industry were the highest in 2017 when compared to the other countries. A study performed by Esposito et al. [50] investigated dioxin emissions from a large sinter plant in Taranto (Italy) over the period of 2007–2013 and concluded that dioxin emissions have significantly reduced from 2008–2013, following the introduction of the Regional Regulation (LR44/2008). Aries et al. [51] studied the emission of the PCDD/Fs from three sinter plants located in United Kingdom (UK). The results indicated that the emissions were approximately 29.5 g WHO-TEQ per annum from the UK sinter plants, with 2, 3, 7, 8 –PCDD/Fs accounting for 27.8 g WHO-TEQ/year [51]. The use of electric arc furnace (EAF) has been widely expanded for steel production over the years [52]. Comparing emission data from the blast furnace (BF) route for iron production to electric arc furnace (EAF) route for steel production located in Australia revealed that emissions from the BF route were significantly higher than the EAF route.

Although production of electricity has increased, emission of dioxins from the public electricity generation sector has significantly reduced over the past two decades. Comparing recent emission data from public electricity generation for EU-28 countries and Australia showed that Australia is the highest producers of dioxins per TJ of generated electricity, followed by Cyprus and Portugal. Lin et al. [53], compared emissions from major dioxin sources, such as secondary aluminium smelting, open burning of rice straw, coal-fired power plants and electric arc furnaces in Taiwan and major metropolitan area. The study concluded that the focus must be shifted from ferrous or non-ferrous metal production or medical and municipal waste incineration to coal-fired power plants, which were the highest emitters of dioxins in Taiwan [53]. Assessing the amount of dioxins emitted from different fuel sources used by power plants in Australia revealed that emissions from coal fired power plants were significantly higher when compared to natural gas power plants. Substitution of coal fired power plants with renewable energy sources, would be a better option to reduce dioxin emissions in Australia.
